# DeepSynth: Three-dimensional nuclear segmentation of biological images using neural networks trained with synthetic data

**DOI:** 10.1038/s41598-019-54244-5

**Published:** 2019-12-04

**Authors:** Kenneth W. Dunn, Chichen Fu, David Joon Ho, Soonam Lee, Shuo Han, Paul Salama, Edward J. Delp

**Affiliations:** 1 Department of Medicine, Division of Nephrology Indiana University School of Medicine, 950 West Walnut St, R2-202, Indianapolis, IN 46202 USA; 2 Video and Image Processing Laboratory, School of Electrical and Computer Engineering, Purdue University, West Lafayette, IN 47907 USA; 3 Department of Electrical and Computer Engineering, Indiana University-Purdue University Indianapolis, Indianapolis, IN 46202 USA

**Keywords:** Fluorescence imaging, Image processing

## Abstract

The scale of biological microscopy has increased dramatically over the past ten years, with the development of new modalities supporting collection of high-resolution fluorescence image volumes spanning hundreds of microns if not millimeters. The size and complexity of these volumes is such that quantitative analysis requires automated methods of image processing to identify and characterize individual cells. For many workflows, this process starts with segmentation of nuclei that, due to their ubiquity, ease-of-labeling and relatively simple structure, make them appealing targets for automated detection of individual cells. However, in the context of large, three-dimensional image volumes, nuclei present many challenges to automated segmentation, such that conventional approaches are seldom effective and/or robust. Techniques based upon deep-learning have shown great promise, but enthusiasm for applying these techniques is tempered by the need to generate training data, an arduous task, particularly in three dimensions. Here we present results of a new technique of nuclear segmentation using neural networks trained on synthetic data. Comparisons with results obtained using commonly-used image processing packages demonstrate that DeepSynth provides the superior results associated with deep-learning techniques without the need for manual annotation.

## Introduction

Over the past 30 years fluorescence microscopy has grown from an approach that was largely descriptive into a truly quantitative technique. Initially founded upon the development of sensitive digital detectors, and then fueled by the development of powerful new microscope designs and fluorescent proteins, quantitative fluorescence microscopy has become a primary tool in biomedical research.

In general, the first step in quantification of fluorescence images is delineation of regions-of-interest, e.g., individual cells or regions within a cell. For studies involving relatively few measurements, this can be easily accomplished by manually outlining regions-of-interest, whose fluorescence can then be measured using available software. However, this approach quickly becomes impractical as the number of measurements increases and/or the regions must be defined in three dimensions. Thus, in most cases, the process of defining regions of interest involves automated image segmentation, a process in which the regions-of-interest are extracted from the images automatically using image analysis/processing techniques.

Automated segmentation of fluorescence images is challenging. Characteristics that are obvious to the human eye are frequently difficult to distill into quantitative features that can be used by a computer to discriminate regions. Standard edge-detection algorithms can be used to discriminate the lateral boundaries of cultured cells grown at low density, but these simple approaches fail to discriminate cells at high density, such as in biological tissues. One common workaround to this problem is to segment nuclei in images collected from tissues, and then characterize each cell from the fluorescence in the surrounding region^1–3^. A variety of novel morphological approaches have been developed to segment nuclei in two-dimensional images collected from cultured cells^4^, in three-dimensional (3D) images collected from cultured cells^5^ and in 3D images collected from biological tissues such as mouse pancreatic islets^6^, rat hippocampus^7^, nematode brain^8^, mouse embryo^9,10^, tumor spheroids^9,11^, cervical tumor^12^ and human breast^13^ and in time-series 3D images of developing zebrafish embryos^14^.

However, images collected in biological microscopy vary wildly with respect to resolution, signal-to-noise ratios, contrast and background. Consequently, image segmentation solutions are seldom robust; approaches optimized for one set of images frequently perform poorly for others. One exciting solution that is essentially designed to adapt to the unique qualities of different images, is based upon deep learning^15,16^, a process in which the characteristic features of objects are derived from the data itself. The user provides a set of training data that includes objects that have been outlined by the user, from which a convolutional neural network derives a set of common features that are then used to discriminate objects in the experimental data.

Deep-learning, which is emerging as a powerful new tool in quantitative biological microscopy^17–19^, has shown great promise as an approach for robust segmentation of biological imaging data^19–25^. However, a potential barrier to widespread adoption of deep-learning for nuclear segmentation is the laborious process of generating training data, which for nuclear segmentation consists of manually outlining the borders of hundreds to thousands of nuclei. The process is especially onerous in 3D, in which the poor axial resolution of optical microscopy makes the top and bottom boundaries difficult to reproducibly delineate.

One approach to reducing the burden of generating training data is to replace hand-annotated images with synthetic images that capture the salient features of the experimental data, but whose boundaries, by definition, are known in advance. This approach has been successfully applied for segmentation of nuclei in two-dimensional images^20,21^. Here we demonstrate DeepSynth, an approach that extends this strategy to 3D. Based upon an approach that we previously described in which neural networks are trained on 3D synthetic data^26,27^, DeepSynth is a fully automated tool for 3D segmentation that provides the robust performance of a deep-learning-based approach without the need for manually-annotated training data. Here we present quantitative comparisons of performance across a range of different fluorescence image volumes, demonstrating that DeepSynth provides accuracy that generally exceeds that provided by available software, while eliminating the need to optimize segmentation parameters for each volume.

## Methods

### Microscope image collection

**Figure 1:**
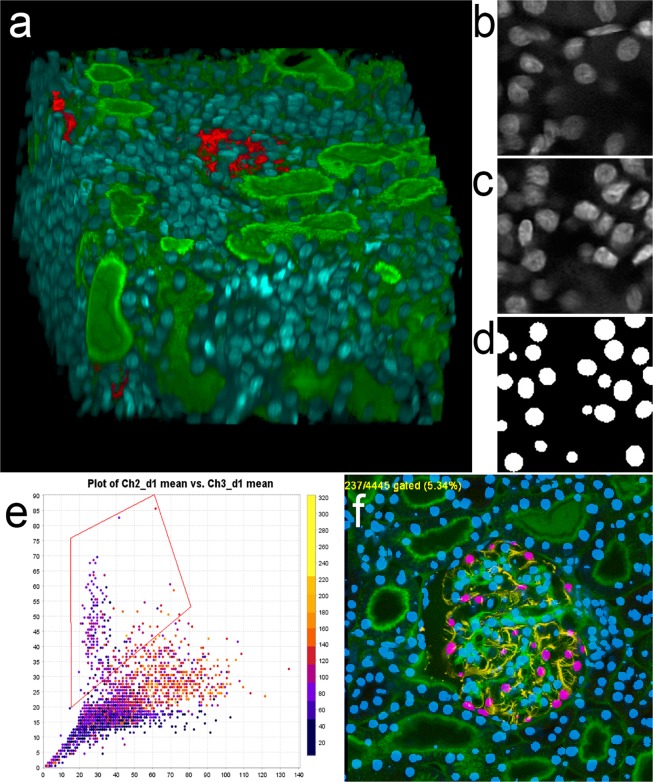
Three-dimensional image collected from cleared kidney sample. (**a**) 3D rendering of an image volume collected from rat kidney tissue following clearing. Red – anti-vimentin immunofluorescence, Green – fluorescein-labeled Lens culinaris agglutinin, Blue – Hoechst 33342-labeled nuclei. A movie of the volume rendering is shown in Supplementary Video 1. (**b**) Single plane image from a subvolume of the image of nuclei that was used to derive the synthetic image volume. (**c**) Single plane image from the synthetic image volume obtained from volume shown in panel B. (**d**) Binary segmentation of the focal plane shown in panel c. (**e**) Screen capture of scatterplot from VTEA, in which the mean fluorescence intensity of fluorescein-Lens culinaris is plotted against the mean fluorescence intensity of an anti-vimentin antibody (x and y, respectively). Box indicates gate used to distinguish podocytes in panel f. (**f**) Screen-capture of image window from VTEA, showing segmented nuclei (blue) and gated podocytes (pink). Image volume shown in panel a is 256 microns across and 144 microns deep. Panels b, c and d represent an image field that is 32 microns across.

**Figure 2:**
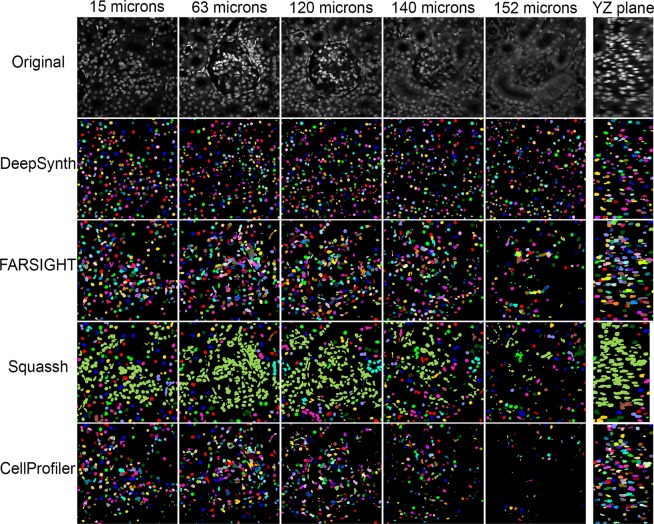
Comparison of segmentations obtained from DeepSynth with those obtained using software commonly used in biomedical imaging. Images collected from different depths of the volume shown in Fig. 1 are shown in the top row. Segmentation results obtained using DeepSynth, FARSIGHT, Squassh and CellProfiler are shown in the rows below. Individual objects are rendered in different colors to facilitate evaluation of discrimination of individual nuclei. Supplementary Videos 2, 3 and 4 show animations of volume renderings of segmentations obtained using DeepSynth with those obtained using FARSIGHT, Squassh and CellProfiler, respectively, for a subvolume ranging from 130 to 162 microns depth in the sample.

**Figure 3:**
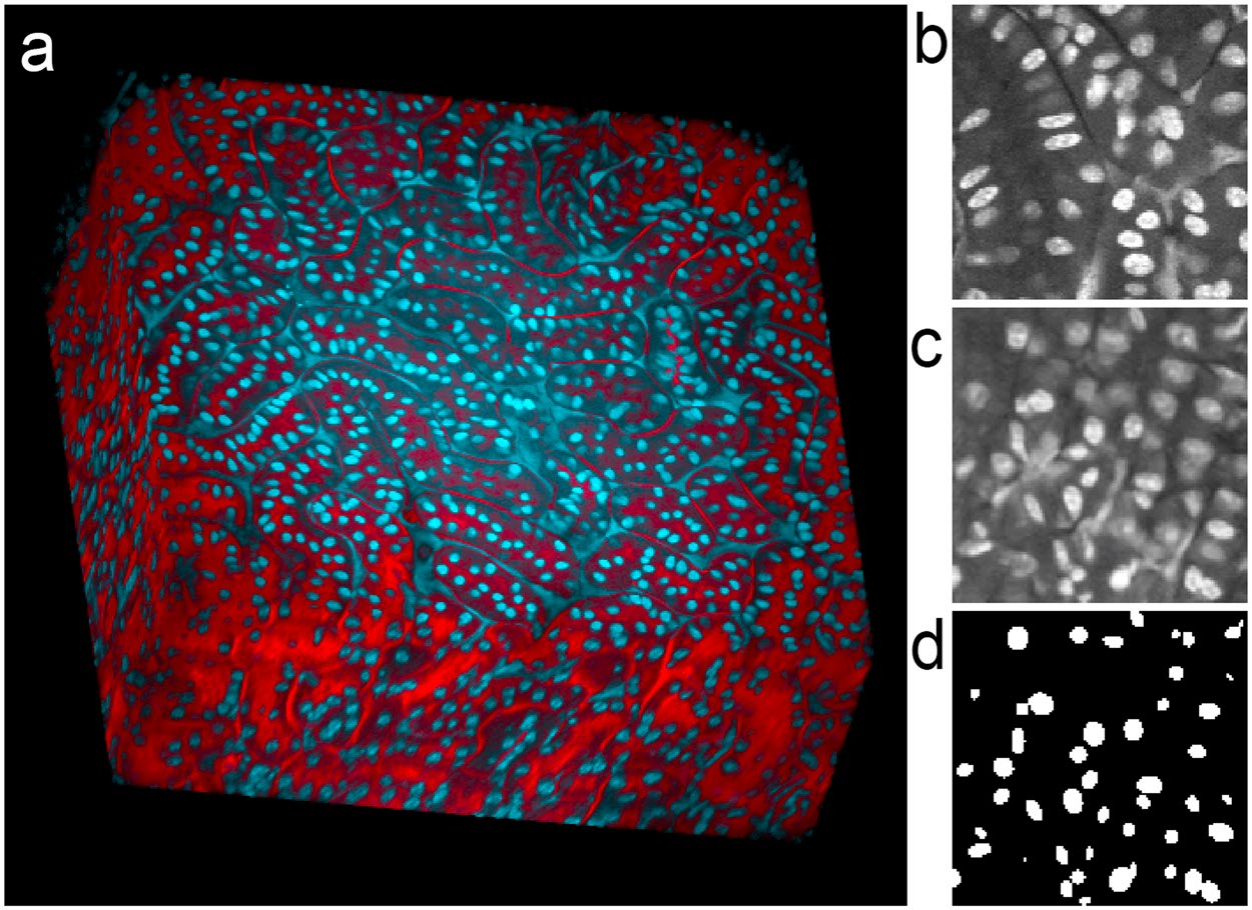
Three-dimensional image collected from cleared kidney sample with non-specific fluorescence. (**a**) Volume rendering of rat kidney tissue following clearing. Red – TexasRed-phalloidin, Blue – Hoechst 33342-labeled nuclei. A movie of the volume rendering is shown in Supplementary Video 5. (**b**) Single plane image from a subvolume of the image of nuclei that was used to derive the synthetic image volume. (**c**) Single plane image from the synthetic image volume obtained from volume shown in panel B. (**d**) Binary segmentation of the focal plane shown in panel c. Image volume shown in panel A is 512 microns across and 200 microns deep. Panels b, c and d represent an image field that is 64 microns across.

**Figure 4:**
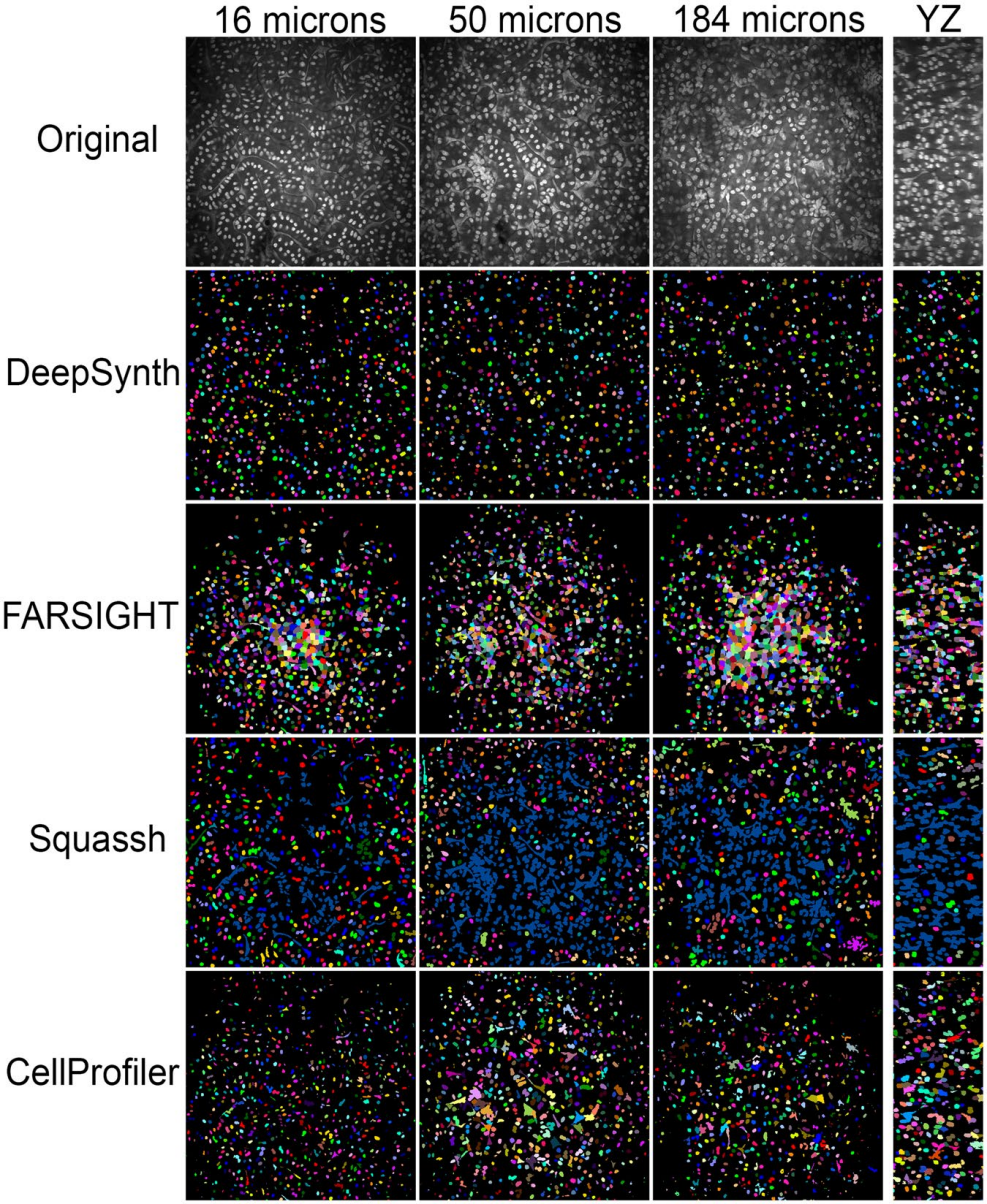
Comparison of segmentations obtained from DeepSynth with those obtained using software commonly used in biomedical imaging. Images collected from different depths of the volume shown in Fig. 3 are shown in the top row. Segmentation results obtained using DeepSynth, FARSIGHT, Squassh and CellProfiler are shown in the rows below. Individual objects are rendered in different colors to facilitate evaluation of discrimination of individual nuclei. Supplementary Videos 6, 7, and 8 show animations of volume renderings of segmentations obtained using DeepSynth with those obtained using FARSIGHT, Squassh and CellProfiler, respectively, for a subvolume ranging from 31 to 50 microns depth in the sample.

**Figure 5:**
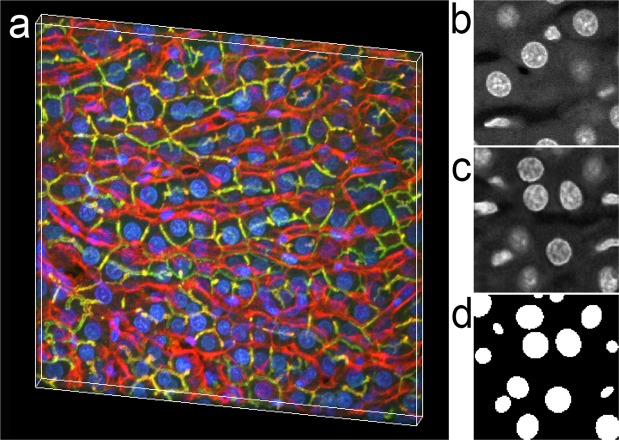
Three-dimensional image collected from fixed rat liver tissue. (**a**) Volume rendering of a thin section of rat liver tissue. Red – TexasRed-phalloidin, Green – Alexa488 anti-Mrp2, Blue – Hoechst 33342-labeled nuclei. A movie of the volume rendering is shown in Supplementary Video 9. (**b**) Single plane image from a subvolume of the image of nuclei that was used to derive the synthetic image volume. (**c**) Single plane image from the synthetic image volume obtained from volume shown in panel B. (**d**) Binary segmentation of the focal plane shown in panel C. Image volume shown in panel A is 256 microns across and 32 microns deep. Panels b, c and d represent an image field that is 32 microns across.

**Figure 6:**
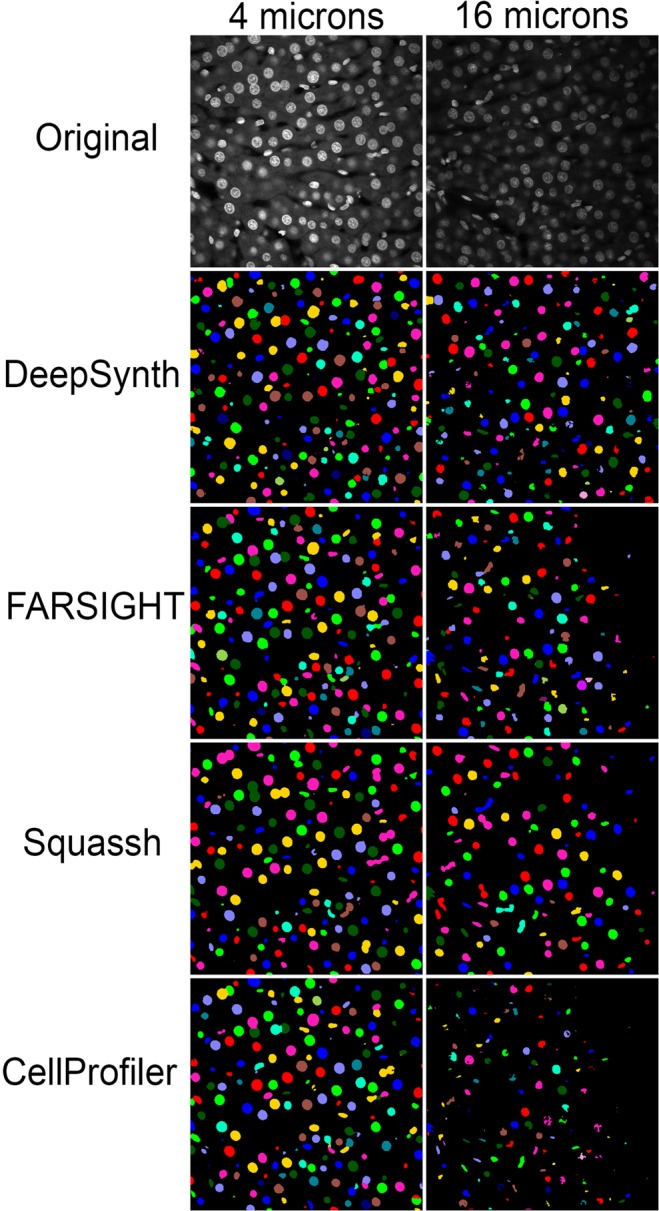
Comparison of segmentations obtained from DeepSynth with those obtained using software commonly used in biomedical imaging. Images collected from different depths of the volume shown in Fig. 5 are shown in the top row. Segmentation results obtained using DeepSynth, FARSIGHT, Squassh and CellProfiler are shown in the rows below. Individual objects are rendered in different colors to facilitate evaluation of discrimination of individual nuclei. Supplementary Videos 10, 11 and 12 show animations of volume renderings of segmentations obtained using DeepSynth with those obtained using FARSIGHT, Squassh and CellProfiler, respectively, for a subvolume extending through the entire depth of the sample.

**Figure 7:**
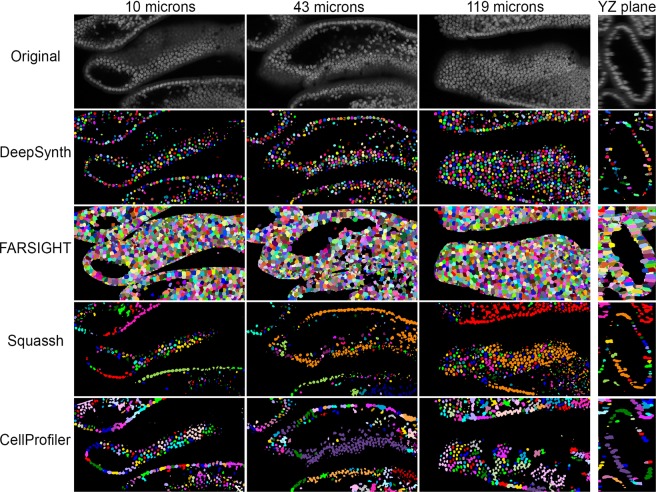
Mouse intestine - comparison of segmentations obtained from DeepSynth with those obtained using software commonly used in biomedical imaging. Images collected from different depths of the volume of nuclei collected from mouse intestine tissue are shown in the top row. Segmentation results obtained using DeepSynth, FARSIGHT, Squassh and CellProfiler are shown in the rows below. Individual objects are rendered in different colors to facilitate evaluation of discrimination of individual nuclei. Each panel is collected from a region of the sample that is 242 microns wide. Supplementary Videos 13, 14 and 15 show animations of volume renderings of segmentations obtained using DeepSynth with those obtained using FARSIGHT, Squassh and CellProfiler, respectively, for a subvolume ranging from 19 to 44 microns depth in the sample.

Images of paraformaldehyde-fixed rat kidney tissue shown in Figs. 1 and 2 were collected with a 40X NA 1.3 oil immersion objective, using an Olympus FV1000 confocal microscope system (Olympus America, Inc., Center Valley, PA, USA) adapted for two-photon microscopy. Rat kidney tissues were fixed, cleared and imaged using confocal microscopy (anti-vimentin immunofluorescence, and Lens culinaris agglutinin) and multiphoton microscopy (Hoechst33342-labeled nuclei) as previously described^28^. An Olympus Fluoview 1000 MPE confocal/multiphoton microscope system mounted on an Olympus IX-81 inverted stand (Olympus America, Inc., Center Valley, PA, USA), equipped with an Olympus 60X oil immersion objective was used to collect images of rat kidney shown in Figs. 3 and 4. For these figures, paraformaldehyde-fixed tissue was labeled with phalloidin and Hoechst 33342, cleared and mounted in Scale mounting medium^29^ and imaged by confocal microscopy using an Olympus 25X, NA1.05 water immersion objective. The same microscope system was used to collect immunofluorescence images of paraformaldehyde-fixed rat liver tissue (phalloidin, anti-Mrp2 immunofluorescence and Hoechst 33342-labeled nuclei) shown in Supplementary Fig. 2 and Figs. 5 and 6. Images of paraformaldehyde-fixed mouse intestine shown in Fig. 7 were labeled with DAPI and imaged using confocal microscopy with a Leica SP8 confocal/multiphoton microscope using a 20X NA 0.75 multi-immersion objective. Tissues were cleared using a modified version of previously described procedures^30^.

### DeepSynth image segmentation

Deepsynth^31^ achieves 3D segmentation and identification of nuclei using machine-learning techniques, in particular deep learning. DeepSynth uses a modified version of U-Net^32^, a 3D convolutional neural network (CNN), for the 3D segmentation of nuclei. The architecture of the DeepSynth CNN (shown in Supplementary Fig. 1) consists of two paths: a down-sampling path and up-sampling path with five layers each, respectively. Each layer consists of two 3D convolutions, batch normalization, and a leaky rectified linear unit activation (Leaky ReLU). The filter size of each 3D convolution is 3 × 3 × 3 with a 1 × 1 × 1 voxel padding such that the output of each convolution step will retain the original volume size. 3D max pooling with a stride of 2 is utilized between the layers in the down-sampling path whereas a 3D transpose convolution is used in the up-sampling path. The objective of this two stage process of down-sampling (achieved through max-pooling) and up-sampling is to enable DeepSynth to extract and learn features that can be used in the segmentation and identification steps. Concatenation is used to transfer information between the down-sampling path and corresponding up-sampling path. At the end of the down-sampling and up-sampling path, a 3D convolution, batch normalization, and 3D sigmoid activation function are used to classify whether each voxel belongs to the foreground (i.e. nuclei) or background (i.e. no nuclei). The output is a 3D binary-valued volume where each voxel indicates where DeepSynth has detected the location of the nuclei.

For “better” learning we use a training loss/cost function for our CNN that is a linear combination of Dice loss (${L}_{DICE}$)^33^ and binary cross-entropy loss (${L}_{BCE}$)^34^:

$${L}_{seg}(T,S)={\mu }_{1}{L}_{Dice}(T,S)+{\mu }_{2}{L}_{BCE}(T,S)$$

where T is the set of ground-truth data, S is a probability map of the 3D binary volumetric segmentation, and ${\mu }_{1}$ and ${\mu }_{2}$ are the weight coefficients for ${L}_{DICE}$ and ${L}_{BCE}$, respectively. The combination of two loss functions improves segmentation performance since ${L}_{DICE}$ constrains the shape of the segmented nuclei while ${L}_{BCE}$ effectively predicts the binary classification (nuclei/no nuclei) of each voxel. As a post-processing step, a 2D watershed^35^ is used sequentially in each of the three orthogonal planes to separate overlapping nuclei in a quasi 3D manner.

The DeepSynth CNN is trained on synthetic data, thus eliminating the need for manually annotated 3D image volumes^26^. We first generate 200 synthetic binary valued 3D volumes by inserting 3D ellipsoid structures, having random rotations and translations. These synthetic binary volumes are used in place of manually annotated volumes where each of the ellipsoid structure represents a single nucleus in the volume. Each volume is constructed such that no two nuclei overlap by more than 5 voxels. The size of each ellipsoid structure is randomly chosen within a preset range corresponding to the characteristics of nuclei in the original 3D volume.

After we generate each synthetic 3D binary volume, we use it with sub-volumes extracted from the original image volumes to train a spatially constrained CycleGAN^36^ (SpCycleGAN) and obtain a generative model that is used to synthesize a synthetic microscopy volume from the synthetic binary volume^26,27,31^. Thus, we now have 200 pairs of synthetic binary volumes (i.e., “the 3D annotations”) and their corresponding synthetic microscopy volumes (i.e, “the original volumes”). We then divide each generated volume into 8 subvolumes, resulting in 1600 pairs of synthetic binary volumes and corresponding synthetic microscopy volumes that are used to train DeepSynth. DeepSynth was implemented in PyTorch using the Adam optimizer^37^ and a learning rate of 0.001. The DeepSynth code is available upon request from Edward J. Delp (ace@ecn.purdue.edu). DeepSynth training and segmentation was conducted using a computer system equipped with an Intel Core i7-6900K 3.2 GHz processor, 128GB RAM and four NVIDIA Titan Xp GPUs, but DeepSynth can be run on a system with as little as 16 GB of RAM and a single GPU (NVIDIA GEFORCE GTX 1080 or similar).

### VTEA image analysis

The use of DeepSynth-segmented nuclei for quantitative tissue cytometry was illustrated using VTEA (Volumetric Tissue Exploration and Analysis) software^3,38^. Segmentation results obtained from DeepSynth were used to define nuclei and fluorescence signal levels of TexasRed (anti-vimentin) and fluorescein (Len agglutinin) were quantified in a region 2 voxels removed from the nuclear border. VTEA provides the ability to define the distance from nuclei at which fluorescence measurements will be obtained, an important feature that can be used to compensate for inaccuracies in the boundaries of the segmented nuclei. For investigators using other 3D image analysis software that samples the voxels immediately surrounding the nuclei, DeepSynth provides the capability to dilate the boundaries of the segmented nuclei, effectively accomplishing the same goal of ensuring sampling outside the boundaries of the nucleus.

### Comparisons of segmentation performance

Segmentation results obtained using DeepSynth were compared with results obtained from CellProfiler 3.0^39^, Squassh^40^, and FARSIGHT^41^, image processing packages that are widely used in biomedical microscopy. In each case, comparisons were made with these tools using either default settings or with settings optimized to the best of our ability, as outlined below.

#### CellProfiler 3.0

CellProfiler segmentations were obtained using both the default settings and settings that were chosen to produce visually “optimal” results on preprocessed images. Typically, CellProfiler works by developing personalized task specific “pipelines” through the addition and arranging of functional modules. The default settings used here constitute a typical pipeline for segmentation using the “IdentifyPrimaryObject” module. The optimized settings were then developed by adding pre-processing and post-processing modules to the default pipeline based on our observations of the results obtained by the default settings. The final settings are chosen from the combination and arrangement of modules that provide the best results.

The inhomogeneity of microscopy images caused the center regions of images to be brighter than the corner regions. Thus, in the default settings results, the darker corner regions are poorly segmented. An illumination correction which uses a sliding window is added as pre-processing to improve the segmentation result of the corner regions. Illumination correction is followed by medium filtering to remove any artifacts caused by the illumination correction step.

Here are the steps we used in the optimized settings. First, we preprocessed the images with the illumination correction step which includes the background correction and 2D median filtering with the“MedianFilter” module for each image in 2D. The window sizes for the illumination correction and 2D median filtering are tuned to achieve the best results.

Secondly, a preprocessing with rescaling image intensity and erosion is done to improve the segmentation result based on our testing. The “RescaleIntensity” module is used to reduce variation from image batches and make the result more reproducible. The “Erosion” module is used to separate touching nuclei. Then, a 3D median filtering with the “MedianFilter” module is done to the image volume to remove any artifacts caused by preprocessing. The window size for the 3D median filter is adjusted to achieve the best results. Note that the best results are determined by visual observation.

Finally, segmentation is done to process the whole volume in 3D with the Otsu’s thresholding^42^ and a 3D watershed^35^ with the “IdentifyPrimaryObject” module. CellProfiler image processing was conducted using a Macbook Pro equipped with an Intel i5 2.6 GHz processor and 8 Gb of RAM.

#### Squassh

For Squassh, we adjusted three parameters to to produce the visually best segmentation results. The first parameter is the “Rolling ball window size” from background subtraction. The default is no background subtraction. We set the window size large enough to have an object within the window. The second parameter is the “Regularization parameter” for segmentation. The default value of the “Regularization parameter” is 0.05. We use higher values to avoid segmenting noise-induced small intensity peaks. The third parameter is “Minimum object intensity” for segmentation. The default value of the “Minimum object intensity“ is 0.15. We use high values to force object separation. Squassh image processing was conducted using a computer system equipped with an Intel core i7-6900K 3.2 GHz CPU and 128 Gb of RAM.

#### FARSIGHT

In the case of FARSIGHT, four parameters σ_min_, σ_max_, r_xy_, and r_z_ are adjusted. Here, σ_min_ and σ_max_ are minimum and maximum scale values of the Laplacian of Gaussian filter. r_xy_ is used to define a search area in which objects are clustered together in the in xy dimension and r_z_ is used to define a search area to create clusters along the z direction. FARSIGHT automatically estimates the values of these four parameters and denotes their values as the default setting. We tested 5 or more combinations of the 4 parameters including the default setting and chose the best-looking segmentation results denoted as an optimized result. FARSIGHT image processing was conducted using a computer system equipped with an Intel core i7-6900K 3.2 GHz CPU and 128 Gb of RAM.

### Quantitative measurement of segmentation performance

Ground truth images of the original (not synthetic) 3D volumes were generated using ITK-SNAP^43^ a commonly used tool for 3D medical image segmentation. Each individual nucleus was manually selected and segmented in 3D using the graphical user interface of ITK-SNAP. The groundtruthing process involved manually labeling voxels of nuclei along all slices in the image volume. Adjacent nuclei were labelled with different colors for better visual representation.

Accuracy was measured using both voxel-based metrics (measuring voxel-by-voxel agreement with ground-truth data) and object-based metrics (measuring agreement in the detection of objects with ground-truth data). Voxel-based accuracy is defined as:

$$VA=\frac{{N}_{tp}^{v}+{N}_{tn}^{v}}{{N}_{total}^{v}},\,Type-I=\frac{{N}_{fp}^{v}}{{N}_{total}^{v}},\,Type-II=\frac{{N}_{fn}^{v}}{{N}_{total}^{v}},$$

where ${N}_{tp}^{v}$, ${N}_{tn}^{v}$, ${N}_{fp}^{v}$, and ${N}_{fn}^{v}\,$are defined as to be the number of segmented voxels that were labeled as true positives, true negatives, false positives, and false negatives, respectively, and ${N}_{total}^{v}$ denotes the total number of voxels in an image. Type-I error (false positive rate) is the ratio of the number of background pixels wrongly detected as nuclei (${N}_{fp}^{v}$) to the ${N}_{total}^{v}$. Similarly, Type-II error (false negative rate) is the ratio of the number of nuclei pixels wrongly detected as background (${N}_{fn}^{v}$) to the ${N}_{total}^{v}$.

Object-based accuracy is measured using the F1 score, which is the harmonic mean of precision (P) and recall (R). The number of segmented nuclei correctly identified as nuclei objects is denoted by ${N}_{tp}^{o}$, the number of segmented nuclei that are non-nuclei but identified as nuclei by ${N}_{fp}^{o}$, and the number of segmented nuclei that are not correctly identified as nuclei by ${N}_{fn}^{o}$, respectively. Then, P and R are obtained as:

$$P=\frac{{N}_{tp}^{o}}{{N}_{tp}^{o}+{N}_{fp}^{o}}{\rm{and}}\,R=\frac{{N}_{tp}^{o}}{{N}_{tp}^{o}+{N}_{fn}^{o}}.$$

Given the value of P and R, the F1 score is obtained as:

$$F1=\frac{2PR}{P+R}.$$

Following a previously described approach^44^, a “true-positive” nucleus is defined as a segmented nucleus that overlaps at least 50% with its corresponding ground-truth nucleus. Otherwise, the segmented nucleus is classified as a false positive. Conversely, a manually annotated nucleus that has no corresponding segmented nucleus is considered as a false negative (${N}_{fn}^{o}$).

### Figures and videos

Volume renderings were constructed using Voxx^45^, videos were prepared using Metamorph (Molecular Devices, Inc) and compressed using TMGEnc (Pegasus, Inc.). Figures were prepared using Photoshop CC 2018 (Adobe, Inc.).

## Results

### Segmentation of nuclei in 3D image volumes collected from thick sections of rat kidney tissue

The DeepSynth segmentation technique was first tested in a 3D fluorescence image volume collected from rat kidney tissue. The image volume consists of 350 images collected to a depth of 175 microns into the tissue. Despite being optically cleared, the thickness of this volume is sufficient to compromise the contrast and resolution of the images collected from the deepest regions of the volume. The nuclei of the tissue were labelled with the DNA-binding probe Hoechst 33342, and the tissue was also labeled with an antibody to vimentin, to label podocytes and with Lens culinaris agglutinin, to label the glycocalyx. The volume was collected using a combination of confocal microscopy (for Lens culinaris agglutinin and TexasRed anti-vimentin) and two-photon microscopy (for Hoechst-labeled nuclei).

Figure 1a shows a volume rendering of the first 124 microns of the resulting image volume, showing green labeling of the glycoproteins on the surface of renal tubules and blue labeling of nuclei throughout the volume. The dense labeling of nuclei obscures all but the very top of a glomerulus (labeled with anti-vimentin immunofluorescence) located in the center of the volume. The interior of the volume can be seen in the animated volume rendering shown in Supplementary Video 1. As described in “Methods” a set of 200 synthetic image volumes were constructed and used for training the DeepSynth CNN. A typical image from the original volume used to derive synthetic data is shown alongside a typical synthetic image and the corresponding DeepSynth segmented image, in Fig. 1b–d, respectively.

Figure 1e,f provide an illustration of how nuclear segmentations can be used as a first step in a quantitative analysis of the cellular constituents of biological tissues. Using the nuclear segmentation provided by DeepSynth, VTEA 3D image analysis software^3^ was used to quantify the amount of fluorescein and TexasRed fluorescence in regions surrounding each of the 4445 nuclei in the volume. VTEA was then used to present these data as a scatterplot and, similar to an analysis of flow cytometry data, to draw a “gated” region on a scatterplot identifying cells high in vimentin, with intermediate levels of Lens culinaris agglutinin. The single image plane shown in Panel F shows that this gating strategy appears to effectively distinguish vimentin-rich podocytes not only from the cells of the surrounding tubular-interstitium, but also from glycocalyx-rich mesangial cells within the glomerulus. Based upon this gating, an analysis of the entire volume indicates that of the 4445 nucleated cells in the volume, 793 are located in the glomerulus and 237 are podocytes. While it is formally possible to conduct a census of this kind manually, the process would be impractically tedious and time-consuming. The largely automated analysis described above was conducted in a matter of minutes.

The results obtained by DeepSynth segmentation at different depths in this volume are shown in Fig. 2. The series of original images displayed at the top of the figure demonstrate that the original images are compromised both by vignetting (reduced signal at the periphery of each image) and by a loss of contrast with depth. Despite these challenges, DeepSynth retrieves nearly all nuclei from all regions of the field at all depths. Figure 2 also shows comparisons with segmentation results obtained using FARSIGHT (http://www.farsight-toolkit.org)^41^, Squassh^40^ and CellProfiler 3.0^39^, three image analysis software packages commonly used by biomedical researchers. Results obtained from FARSIGHT, Squassh and CellProfiler reflect workflows in which images were pre-processed and segmentation parameters adjusted to provide the best results, as evaluated visually (see “Methods”). Insofar as DeepSynth derives object features from the data itself, it requires no adjustment of segmentation parameters. Since evaluations of 3D segmentations in single planes can be misleading, we also present comparisons in the form of 3D animations of volume renderings. Supplementary videos 2, 3 and 4 show side-by-side volume renderings of the DeepSynth segmented volume, the original volume, and the segmented volumes obtained from the alternative software with and without optimization.

Results obtained from FARSIGHT and CellProfiler were similar to those obtained from DeepSynth at shallow depths, but the number of nuclei detected declined with depth, particularly for CellProfiler. Squassh likewise failed to detect many of the nuclei detected at depth by DeepSynth, but suffered a more pervasive problem in distinguishing individual nuclei, instead detecting a single object consisting of hundreds of individual nuclei.

In many cases, the differences in performance are difficult to evaluate visually. In order to quantitatively compare the segmentation performance of DeepSynth with that of FARSIGHT, Squassh and CellProfiler, we quantified accuracy for each, based upon comparisons with a hand-segmented 64 by 64 by 64 voxel subregion of the volume. As described in “Methods”, accuracy was evaluated using voxel-based metrics (agreement in the definition of object boundaries) and object-based metrics (agreement in the identification of each object in the volume, without regard to the accuracy of its boundaries).

The results of these analyses, shown in Table 1, demonstrate that all techniques perform well with respect to voxel-based accuracy at both depths of the volume, reflecting excellent performance in accurately distinguishing the boundaries of nuclei. The discrepancy between these high accuracy measurements and the variable results shown in Fig. 2 reflect the shortcomings of voxel-based accuracy measurements; they are relatively insensitive to failures to detect or discriminate individual nuclei, a factor that is critical to the overall goal of characterizing individual cells in a tissue. The results of the object-based analysis are more consonant with the visual appearance of the segmentations. The ability of DeepSynth to detect and discriminate nuclei throughout the volume is reflected in high F1 scores (the harmonic mean of precision and recall) at both depths. In contrast, the inability of Squassh to distinguish individual nuclei resulted in the low F1 scores at both depths of the volume. F1 scores obtained for segmentations generated by CellProfiler were reasonably high in the shallow volume, but declined at depth. FARSIGHT generated the highest F1 score of any technique at shallow depths, but its performance declined in the deeper volume.

**Table 1:** Quantitative analysis of segmentation results obtained from volume shown in Figs. 1 and 2. Segmentation results obtained from DeepSynth were quantitatively compared with those obtained from FARSIGHT, Squassh and CellProfiler using either default settings or settings optimized as described in “Methods”.

Segmentation technique	Time (entire volume)	Voxel based			Object based		
		Type I	Type II	Accuracy	Precision	Recall	F1
		**Sub-volume collected 75–112 microns from the surface**					
DeepSynth	94 sec	4.03%	3.81%	92.15%	72.80%	90.55%	80.71%
FARSIGHT Default	13 min	9.61%	0.92%	89.47%	65.94%	94.62%	77.72%
FARSIGHT Optimized	13 min	9.55%	1.01%	89.44%	78.09%	87.11%	82.53%
Squassh Default	Hours	9.56%	0.39%	90.05%	92.94%	33.19%	48.92%
Squassh Optimized	Hours	11.45%	0.36%	88.19%	90.41%	27.62%	42.31%
CellProfiler Default	15 min	7.15%	2.02%	90.83%	80.12%	58.37%	67.54%
CellProfiler Optimized	15 min	5.36%	3.06%	91.58%	71.04%	78.89%	74.76%
Otsu-3DWatershed	54 sec	8.99%	1.43%	89.58%	90.58%	52.52%	66.49%
		**Sub-volume collected 130–162 microns from the surface**					
DeepSynth	94 sec	3.24%	4.34%	92.42%	72.94%	92.54%	81.58%
FARSIGHT Default	13 min	4.07%	5.05%	90.88%	43.18%	67.86%	52.78%
FARSIGHT Optimized	13 min	4.08%	5.04%	90.88%	78.95%	68.18%	73.17%
Squassh Default	Hours	8.64%	2.63%	88.73%	83.33%	35.21%	49.50%
Squassh optimized	Hours	3.80%	4.71%	91.49%	76.47%	39.39%	52.00%
CellProfiler Default	15 min	1.30%	7.35%	91.35%	55.32%	48.15%	51.49%
CellProfiler Optimized	15 min	0.46%	10.95%	88.59%	28.57%	26.09%	27.27%
Otsu-3DWatershed	54 sec	3.76%	5.53%	90.71%	62.50%	40.98%	49.50%

The values for “Time” reflect the times required to obtain segmentations using. Accuracy was measured using both voxel-based metrics (voxel-by-voxel agreement with ground-truth data) and object-based metrics (agreement in the detection of objects with ground-truth data) in 64 by 64 by 64 voxel sub-volumes obtained 75–112 microns from the surface of the sample (top) and 130–162 microns from the surface of the sample (bottom). For voxel-based accuracy, type-I error (false positive rate) represents the fraction of voxels in the volume wrongly detected as belonging to nuclei and type-II error (false negative rate) represents the fraction of voxels wrongly detected as background. Object-based accuracy is measured using the F1 score, which is the harmonic mean of precision and recall, where precision is the ratio of the number of correctly identified nuclei to the sum of the number of correctly identified nuclei plus the number of objects incorrectly identified as nuclei and recall is the ratio of the number of correctly identified nuclei to the sum of the number of correctly identified nuclei plus the number of nuclei that failed to be detected. Details of the analyses are described in “Methods”.

These quantitative analyses also demonstrate the difficulty of optimizing segmentation parameters. As discussed above, “optimized” segmentations presented for FARSIGHT, Squassh and CellProfiler reflect hours of effort to identify image preprocessing and segmentation parameter settings yielding the best results, as evaluated visually. In some cases, these adjustments quantitatively improved segmentation performance, but in others they had little effect, or even decreased accuracy scores. In the case of CellProfiler, adjustments that improved performance in the shallow volume profoundly reduced performance in the deep volume.

### Segmentation of nuclei in 3D image volumes of rat kidney containing non-specific fluorescence

One the strengths of deep-learning is the capability to develop discriminatory criteria that aren’t necessarily obvious to the human observer. Figure 3a and Supplementary video 5 show a rendering of an image volume collected from cleared rat kidney tissue labeled with TexasRed-phalloidin (labeling actin red) and Hoechst 33342. While the DNA binding probe Hoechst 33342 strongly labels nuclei, the images also included fluorescence from unbound probe in the vasculature. This vascular probe can be seen as the triangular/filamentous labeling in the volume rendering, as well as in the magnified image of the region used to develop synthetic data and in the image of synthetic data (Fig. 3b,c, respectively).

Segmentations of this volume produced by DeepSynth, FARSIGHT, Squassh and CellProfiler are shown in Fig. 4 (and in Supplementary videos 6, 7 and 8). Whereas DeepSynth accurately discriminated nuclei from non-specific Hoechst fluorescence throughout the volume, the other image software struggled to varying degrees. In the case of FARSIGHT, the inability to discriminate nuclei resulted in a large number of spurious objects. Squassh likewise failed to distinguish nuclei from non-specific fluorescence, aggravating its inability to discriminate adjacent objects resulting in a single object that extended throughout the volume. Of the three alternatives, CellProfiler was the least impacted by the non-specific fluorescence, detecting fewer spurious objects than FARSIGHT, while being more effective at discriminating objects than Squassh.

These visual impressions are borne out in the quantitative analyses of accuracy, shown in Table 2. DeepSynth’s overall segmentation accuracy is markedly higher than that of the alternative approaches, according to either voxel-based or object-based criteria. DeepSynth’s superior ability to discriminate nuclei is evident in a Type I error (false-positive rate) that is 7 to 14 fold lower than any of the other segmentations. Effects on object-based accuracy are even more impressive, although the effects of the non-specific fluorescence vary between software. In the case of FARSIGHT, which sensitively detects both real and spurious objects, and readily discriminates adjacent objects, the spurious objects compromise precision, with no effect on recall. In contrast, Squassh likewise detects both real and spurious objects, but cannot distinguish them, resulting in high levels of precision, but very low recall. Of the alternative software, CellProfiler performed best overall, particularly when optimized.

**Table 2:** Quantitative analysis of segmentation results obtained from volume shown in Figs. 3 and 4. Segmentation results obtained from DeepSynth were quantitatively compared with those obtained from FARSIGHT, Squassh and CellProfiler using either default settings or settings optimized as described in “Methods”.

	Time (entire volume)	Voxel based			Object based		
		Type I	Type II	Accuracy	Precision	Recall	F1
DeepSynth	102 sec	2.14%	1.88%	95.98%	89.38%	95.26%	92.23%
FARSIGHT Default	13 min	30.33%	0.00%	69.67%	64.34%	97.53%	77.53%
FARSIGHT Optimized	13 min	28.58%	0.53%	70.90%	37.72%	97.73%	54.43%
Squassh Default	Hours	24.75%	0.00%	75.25%	90.38%	16.61%	28.06%
Squassh Optimized	Hours	19.54%	0.01%	80.45%	85.07%	20.14%	32.57%
CellProfiler Default	15 min	21.67%	0.31%	78.02%	80.82%	20.92%	33.24%
CellProfiler Optimized	15 min	14.94%	0.11%	84.95%	81.93%	72.08%	76.69%
Otsu- 3DWatershed	32 sec	17.88%	0.23%	81.89%	87.28%	53.36%	66.23%

The values for “Time” reflect the times required to obtain segmentations using. Accuracy was measured using both voxel-based metrics (voxel-by-voxel agreement with ground-truth data) and object-based metrics (agreement in the detection of objects with ground-truth data) in a 64 by 64 by 64 voxel sub-volume. For voxel-based accuracy, type-I error (false positive rate) represents the fraction of voxels in the volume wrongly detected as belonging to nuclei and type-II error (false negative rate) represents the fraction of voxels wrongly detected as background. Object-based accuracy is measured using the F1 score, which is the harmonic mean of precision and recall, where precision is the ratio of the number of correctly identified nuclei to the sum of the number of correctly identified nuclei plus the number of objects incorrectly identified as nuclei and recall is the ratio of the number of correctly identified nuclei to the sum of the number of correctly identified nuclei plus the number of nuclei that failed to be detected. Details of the analyses are described in “Methods”.

### Segmentation of nuclei in shallow 3D image volumes collected from rat liver tissue

The first two examples presented above emphasize the challenges of segmenting image volumes collected deep into biological tissues. However, the microscopy conducted by most biomedical researchers seldom extends much beyond a single layer of cells, either in tissue or grown in culture. In order to evaluate the performance of DeepSynth for segmenting the kinds of images volumes that are more commonly encountered in biological microscopy, we conducted a comparative analysis of a 32 micron thick section of uncleared rat liver tissue. Figure 5 and Supplementary video 9 show a rendering of a 3D volume collected from rat liver tissue labeled with fluorescent phalloidin (red) an antibody to Mrp2 (green) and Hoechst 33342 (blue).

Images of individual planes collected from this volume, shown in Fig. 6, show that contrast rapidly decreases with depth into this uncleared tissue, demonstrating that even thin volumes can present challenges similar to those encountered at much greater depths in cleared tissues. Nonetheless, the comparisons shown in Fig. 6 show that all of the approaches produced results that were visually similar. Voxel-based accuracy scores were likewise high for all approaches (Table 3). However, object-based accuracy was markedly higher for DeepSynth. Examination of Supplementary videos 10, 11 and 12 suggest that this difference may reflect better performance in distinguishing closely-packed nuclei.

**Table 3:** Quantitative analysis of segmentation results obtained from volume shown in Figs. 5 and 6. Segmentation results obtained from DeepSynth were quantitatively compared with those obtained from FARSIGHT, Squassh and CellProfiler using either default settings or settings optimized as described in “Methods”.

	Time (entire volume)	Voxel based			Object based		
		Type I	Type II	Accuracy	Precision	Recall	F1
DeepSynth	37 sec	5.92%	1.79%	92.30%	87.09%	98.45%	92.42%
FARSIGHT Default	26 sec	4.21%	1.72%	94.07%	64.69%	91.92%	75.94%
FARSIGHT Optimized	26 sec	4.18%	1.78%	94.04%	86.45%	92.58%	89.41%
Squassh Default	10 min	4.46%	0.70%	94.84%	91.70%	75.47%	82.79%
Squassh Optimized	10 min	3.19%	1.31%	95.50%	94.49%	74.53%	83.33%
CellProfiler Default	10 min	2.66%	3.39%	93.95%	85.49%	73.65%	79.13%
CellProfiler Optimized	10 min	2.76%	2.34%	94.90%	83.49%	89.33%	86.31%
Otsu- 3DWatershed	29 sec	2.53%	3.51%	93.95%	82.59%	76.37%	79.36%

The values for “Time” reflect the times required to obtain segmentations using. Accuracy was measured using both voxel-based metrics (voxel-by-voxel agreement with ground-truth data) and object-based metrics (agreement in the detection of objects with ground-truth data) for the entire 3D volume. For voxel-based accuracy, type-I error (false positive rate) represents the fraction of voxels in the volume wrongly detected as belonging to nuclei and type-II error (false negative rate) represents the fraction of voxels wrongly detected as background. Object-based accuracy is measured using the F1 score, which is the harmonic mean of precision and recall, where precision is the ratio of the number of correctly identified nuclei to the sum of the number of correctly identified nuclei plus the number of objects incorrectly identified as nuclei and recall is the ratio of the number of correctly identified nuclei to the sum of the number of correctly identified nuclei plus the number of nuclei that failed to be detected. Details of the analyses are described in “Methods”.

### Segmentation of nuclei in 3D image volumes using a network trained on data derived from a different image volume

The results here raise the question of how well a CNN trained on synthetic data derived from one volume performs for segmentation of an unrelated volume. In order to evaluate this question, we applied the CNN used to segment the image volume shown in Fig. 1 to a large image volume collected from cleared mouse intestine tissue. This image volume was noteworthy for the high density of nuclei and relatively poor axial resolution. The results, shown in Fig. 7 and Supplementary videos 13, 14 and 15 show that despite being trained on synthetic volumes derived from a different image volume, DeepSynth sensitively detected and discriminated nuclei throughout the volume. In contrast, both Squassh and CellProfiler struggled with the density of nuclei in this volume, identifying objects consisting of vast networks of unresolved nuclei. Despite an apparent inability to clearly distinguish the boundaries of nuclei, FARSIGHT nonetheless was much more successful at distinguishing adjacent nuclei. Segmentation performance for this volume was not quantified, due to the difficulty that we encountered in trying to reproducibly hand-outline the poorly-defined, densely distributed nuclei.

## Discussion

With the development of digital detectors and methods of digital image analysis, fluorescence microscopy has been transformed from a relatively subjective technique into a quantitative technique. However, the approaches used for quantitative digital image analysis are now challenged by the enormous increase in the volume and complexity of fluorescence microscopy data.

Once limited to two-dimensional images of relatively thin specimens, fluorescence microscopy was extended into three dimensions with the development of confocal microscopy, supporting 3D imaging of cells. The third dimension was subsequently extended from microns to millimeters with the development of two-photon microscopy, light sheet microscopy and the renewed development of methods of tissue clearing. The lateral scale of high-resolution microscopy was extended with the development of automated microscope systems such that it is now possible to collect 3D images of entire organs. The scale of microscopy is expanded still further in temporal studies that involve the collection of multiple image volumes over time. In parallel, the complexity of biological images has been expanded with the development of multiplexing techniques that make it possible to image more than 40 different target molecules in the same sample volume.

These new techniques have made it possible to collect image volumes of unprecedented data richness. However, extracting the information embedded in images of this size and complexity depends upon the development of new methods of automated digital analysis^1–3,46^. The first step in automated digital image analysis is the delineation of the regions that are to be quantified which, in the case of large-scale microscopy are typically individual cells. In most samples, the boundaries of individual cells are difficult, if not impossible to distinguish in tissues (but see^32,46,47^), so that most studies employ a strategy in which individual cells are initially identified by their nuclei. Characterizations of the cells are then based upon fluorescence measurements made in regions surrounding the nuclei.

Nuclei are well-suited to automated segmentation as they can be brightly labeled with either DNA-intercalating probes or genetically expressed fluorescent protein chimeras, resulting in fluorescence images that have clearly defined borders. For this reason, nuclear segmentation has been used as a first step in large-scale quantitative analyses of the cellular constitution of tissues, a procedure termed “3D tissue cytometry”^2,3^ or “3D histo-cytometry”^1^. These studies demonstrate an effective approach for extracting quantitative data from the complex and rich 3D image volumes collected from large tissue samples, supporting comprehensive characterizations of hundreds of thousands of cells in animal tissue samples and biopsies.

While nuclei are easier to segment than cells, they still present challenges to segmentation. First, the cell density in some tissues is so high that the images of the nuclei overlap with one another, making discrimination of individual nuclei more difficult. Second, due to the poorer axial resolution of microscope images, the upper and lower boundaries of nuclei can be difficult to distinguish, compromising 3D segmentation. Finally, due to the cumulative effects of optical aberrations and scattering of light in tissues, image contrast decreases with depth of imaging, so that segmentation approaches that are successful in the shallowest regions of an image volume fail in the deepest regions. Each of these issues need to be addressed in order for image cytometry to realize its full potential as a tool in biomedical research. As a consequence, image segmentation is an active field of research in biological microscopy.

The challenge of segmentation is compounded by the variability in images collected in biological microscopy. The noise, background, contrast and resolution differences between images collected from different tissues, with different microscopes using different settings are such that conventional segmentation approaches developed for one set of studies perform poorly in other studies. The issue of image variability is obviated in segmentation approaches based upon deep-learning, which derive the characteristic features of objects from the sample images themselves.

The major drawback of deep-learning techniques is that the quality of the results depends upon the amount and quality of manually annotated training data. In the case of nuclear segmentation, training data is generated by manually outlining individual nuclei, a tedious process, particularly in 3D. Perhaps for this reason, deep learning approaches have been largely limited to segmentation of nuclei in 2D images^21–25^. The burden of manual annotation of 3D data was recently addressed by the Ronneberger laboratory^32^, who describe a process of “sparse annotation”, a process in which 3D objects are not completely circumscribed in the training data but rather are delineated in selected orthogonal slices.

Here we demonstrate 3D nuclear segmentation using a convolutional neural network trained on synthetic 3D data, obviating the need for manually annotated training data. Quantitative analyses demonstrate that DeepSynth generates nuclear segmentations that equal or surpass the accuracy of segmentations obtained using existing software, particularly under challenging conditions such as in images collected at depth, or from tissues with high nuclear density. Significantly, accurate segmentations were obtained for a range different kinds of images without the need to tune segmentation parameters for each.

The freedom from the need for optimization is an under-appreciated virtue of deep-learning-based segmentation. The process of optimization is time-consuming and susceptible to bias. We also found it to be frustratingly unpredictable. In some cases, hours of effort would be spent to identify settings yielding the most visually-satisfying results, only to find that “optimizing” the settings actually compromised quantitative measures of accuracy. Ideally, segmentation parameters would be optimized for quantitative measures of accuracy, but in practice, few investigators will invest the time to generate the ground-truth data necessary for accuracy measures. Moreover, even optimizing to a quantitative metric will not guarantee success; in some cases we found that adjustments that improved segmentations in some regions of a volume actually compromised segmentations in other regions.

The results presented in Fig. 7 demonstrate that satisfactory segmentations can be obtained using networks trained on different image volumes. In general, while superior results are obtained from networks specifically trained for a given volume, we have found that a network trained on a single volume can be effectively applied to segmentation of additional volumes that are similar with respect to the voxel dimensions and texture of the nuclei. Thus, a single network might be sufficient for a typical study that involves comparisons of multiple samples prepared and imaged in the same way. We are currently exploring this approach. We are also developing new SpCycleGAN training approaches designed to accommodate differences in the size and shape of nuclei which we believe may underlie a few cases where false-negative voxel-based errors were somewhat elevated relative to the other approaches. Finally, we are also examining transfer learning to extend our approaches to different types of cellular types and structures.

## Supplementary information

Supplementary Information

Video 1

Video 2

Video 3

Video 4

Video 5

Video 6

Video 7

Video 8

Video 9

Video 10

Video 11

Video 12

Video 13

Video 14

Video 15

## Data Availability

The DeepSynth code is available upon request from Edward J. Delp (ace@ecn.purdue.edu). Original and segmented image volumes may be obtained from Ken Dunn (kwdunn@iu.edu).

## References

[1] Gerner MY, Kastenmuller W, Ifrim I, Kabat J, Germain RN (2012). Histo-cytometry: a method for highly multiplex quantitative tissue imaging analysis applied to dendritic cell subset microanatomy in lymph nodes. Immunity.

[2] Micanovic R (2018). Tamm-Horsfall Protein Regulates Mononuclear Phagocytes in the Kidney. J Am Soc Nephrol.

[3] Winfree S (2017). Large-scale 3-dimensional quantitative imaging of tissues: state-of-the-art and translational implications. Transl Res.

[4] Molnar C (2016). Accurate Morphology Preserving Segmentation of Overlapping Cells based on Active Contours. Sci Rep.

[5] Gertych A, Ma Z, Tajbakhsh J, Velasquez-Vacca A, Knudsen BS (2016). Rapid 3-D delineation of cell nuclei for high-content screening platforms. Comput Biol Med.

[6] Tran Thi Nhu H, Arrojo EDR, Berggren PO, Boudier T (2017). A novel toolbox to investigate tissue spatial organization applied to the study of the islets of Langerhans. Sci Rep.

[7] Lin G (2003). A hybrid 3D watershed algorithm incorporating gradient cues and object models for automatic segmentation of nuclei in confocal image stacks. Cytometry A.

[8] Toyoshima Y (2016). Accurate Automatic Detection of Densely Distributed Cell Nuclei in 3D Space. PLoS Comput Biol.

[9] Mathew B (2015). Robust and automated three-dimensional segmentation of densely packed cell nuclei in different biological specimens with Lines-of-Sight decomposition. BMC Bioinformatics.

[10] Lou X, Kang M, Xenopoulos P, Munoz-Descalzo S, Hadjantonakis AK (2014). A rapid and efficient 2D/3D nuclear segmentation method for analysis of early mouse embryo and stem cell image data. Stem Cell Reports.

[11] Boutin ME (2018). A high-throughput imaging and nuclear segmentation analysis protocol for cleared 3D culture models. Sci Rep.

[12] Wahlby C, Sintorn IM, Erlandsson F, Borgefors G, Bengtsson E (2004). Combining intensity, edge and shape information for 2D and 3D segmentation of cell nuclei in tissue sections. J Microsc.

[13] Nandy K, Chellappa R, Kumar A, Lockett SJ (2016). Segmentation of Nuclei From 3D Microscopy Images of Tissue via Graphcut Optimization. I.E.E.E. Journal of Selected Topics in Signal Processing.

[14] Stegmaier J (2014). Fast segmentation of stained nuclei in terabyte-scale, time resolved 3D microscopy image stacks. PLoS One.

[15] LeCun Y, Bengio Y, Hinton G (2015). Deep learning. Nature.

[16] Krizhevsky A, Sutskever I, Hinton GE (2017). ImageNet Classification with Deep Convolutional Neural Networks. Commun Acm.

[17] Xing FY, Xie YP, Su H, Liu FJ, Yang L (2018). Deep Learning in Microscopy Image Analysis: A Survey. I.E.E.E. Transactions on Neural Networks and Learning Systems.

[18] Ching Travers, Himmelstein Daniel S., Beaulieu-Jones Brett K., Kalinin Alexandr A., Do Brian T., Way Gregory P., Ferrero Enrico, Agapow Paul-Michael, Zietz Michael, Hoffman Michael M., Xie Wei, Rosen Gail L., Lengerich Benjamin J., Israeli Johnny, Lanchantin Jack, Woloszynek Stephen, Carpenter Anne E., Shrikumar Avanti, Xu Jinbo, Cofer Evan M., Lavender Christopher A., Turaga Srinivas C., Alexandari Amr M., Lu Zhiyong, Harris David J., DeCaprio Dave, Qi Yanjun, Kundaje Anshul, Peng Yifan, Wiley Laura K., Segler Marwin H. S., Boca Simina M., Swamidass S. Joshua, Huang Austin, Gitter Anthony, Greene Casey S. (2018). Opportunities and obstacles for deep learning in biology and medicine. Journal of The Royal Society Interface.

[19] Gupta A (2019). Deep Learning in Image Cytometry: A Review. Cytometry A.

[20] Sadanandan SK, Ranefall P, Le Guyader S, Wahlby C (2017). Automated Training of Deep Convolutional Neural Networks for Cell Segmentation. Sci Rep.

[21] Caicedo, J. C. *et al*. *Evaluation of Deep Learning Strategies for Nucleus Segmentation in Fluorescence Images* (2019). 10.1002/cyto.a.23863 PMC6771982 31313519

[22] Ronneberger O, Fischer P, Brox T (2015). U-Net: Convolutional Networks for Biomedical Image Segmentation. Lect Notes Comput Sc.

[23] Nandy K (2012). Automatic segmentation and supervised learning-based selection of nuclei in cancer tissue images. Cytom Part A.

[24] Kraus OZ, Ba JL, Frey BJ (2016). Classifying and segmenting microscopy images with deep multiple instance learning. Bioinformatics.

[25] Bohm, A., Ucker, A., Jager, T., Ronneberger, O. & Falk, T. ISOODL: Instance Segmentation of Overlapping Biological Objects Using Deep Learning. *I S Biomed Imaging*, 1225–1229, 10.1109/ISBI.2018.8363792 (2018).

[26] Fu, C. C. *et al*. Nuclei Segmentation of Fluorescence Microscopy Images Using Convolutional Neural Networks. *Proceedings of 2017 IEEE International Symposium on Biomedical Imaging (ISBI)*, 704–708, 10.1109/ISBI.2017.7950617 (2017).

[27] Ho, D. J., Fu, C. C., Salama, P., Dunn, K. W. & Delp, E. J. Nuclei Segmentation of Fluorescence Microscopy Images Using Three Dimensional Convolutional Neural Networks. *Proceedings 2017 IEEE Conference on Computer Vision and Pattern Recognition Workshops (CVPRW)*, 834–842, 10.1109/Cvprw.2017.116 (2017).

[28] Clendenon SG, Young PA, Ferkowicz M, Phillips C, Dunn KW (2011). Deep tissue fluorescent imaging in scattering specimens using confocal microscopy. Microsc Microanal.

[29] Hama H (2011). Scale: a chemical approach for fluorescence imaging and reconstruction of transparent mouse brain. Nat Neurosci.

[30] Susaki EA (2014). Whole-brain imaging with single-cell resolution using chemical cocktails and computational analysis. Cell.

[31] Fu, C. C. *et al*. Three Dimensional Fluorescence Microscopy Image Synthesis and Segmentation. *Proceedings 2018 IEEE Conference on Computer Vision and Pattern Recognition Workshops (CVPRW)*, 2302–2310, 10.1109/Cvprw.2018.00298 (2018).

[32] Falk T (2019). U-Net: deep learning for cell counting, detection, and morphometry. Nat Methods.

[33] Milletari, F., Navab, N. & Ahmadi, S. A. V-Net: Fully Convolutional Neural Networks for Volumetric Medical Image Segmentation. *Int Conf 3d Vision*, 565–571, 10.1109/3dv.2016.79 (2016).

[34] Long, J., Shelhamer, E. & Darrell, T. Fully Convolutional Networks for Semantic Segmentation. *Proc 2015 IEEE Conference on Computer Vision and Pattern Recognition (Cvpr)*, 3431–3440, 10.1109/CVPR.2015.7298965 (2015). 10.1109/TPAMI.2016.2572683 27244717

[35] Meyer F (1994). Topographic Distance and Watershed Lines. Signal Process.

[36] Zhu, J. Y., Park, T., Isola, P. & Efros, A. A. Unpaired Image-to-Image Translation using Cycle-Consistent Adversarial Networks. *Proc 2017 IEEE International Conference on Computer Vision (ICCV)*, 2242–2251, 10.1109/Iccv.2017.244 (2017).

[37] Kingma, D. P. & Ba, J. *Adam: A method for stochastic optimization*, (https://arxiv.org/abs/1412.6980, 2017).

[38] Winfree S (2017). Quantitative Three-Dimensional Tissue Cytometry to Study Kidney Tissue and Resident Immune Cells. J Am Soc Nephrol.

[39] McQuin C (2018). CellProfiler 3.0: Next-generation image processing for biology. PLoS Biol.

[40] Rizk A (2014). Segmentation and quantification of subcellular structures in fluorescence microscopy images using Squassh. Nat Protoc.

[41] Al-Kofahi Y, Lassoued W, Lee W, Roysam B (2010). Improved Automatic Detection and Segmentation of Cell Nuclei in Histopathology Images. IEEE T Bio-Med Eng.

[42] Otsu N (1979). A threshold selection method from gray-scale histograms. IEEE transactions on systems, man and cybernetics.

[43] Yushkevich PA (2006). User-guided 3D active contour segmentation of anatomical structures: significantly improved efficiency and reliability. Neuroimage.

[44] Sirinukunwattana K (2017). Gland segmentation in colon histology images: The glas challenge contest. Med Image Anal.

[45] Clendenon JL, Phillips CL, Sandoval RM, Fang S, Dunn KW (2002). Voxx: a PC-based, near real-time volume rendering system for biological microscopy. Am J Physiol Cell Physiol.

[46] Li W, Germain RN, Gerner MY (2017). Multiplex, quantitative cellular analysis in large tissue volumes with clearing-enhanced 3D microscopy (Ce3D). Proc Natl Acad Sci USA.

[47] Baggett D, Nakaya MA, McAuliffe M, Yamaguchi TP, Lockett S (2005). Whole cell segmentation in solid tissue sections. Cytometry A.

