# Supplementary material for: DeepSynth: Three-dimensional nuclear segmentation of biological images using neural networks trained with synthetic data
Source: Sci Rep. 2019 Dec 4;9:18295. doi: 10.1038/s41598-019-54244-5 (PMC6892824; doi:10.1038/s41598-019-54244-5)
Supplement: Supplementary file 1 — Supplementary Information [file 41598_2019_54244_MOESM1_ESM.pdf]

Supplementary Information:

Supplementary Figures
Legends to Supplementary Videos

DeepSynth: Three-dimensional nuclear segmentation of biological images using neural networks trained with synthetic data

Kenneth W. Dunn[1]*, Chichen Fu[2], David Joon Ho[2], Soonam Lee[2], Shuo Han[2], Paul Salama[3] and Edward J. Delp[2]*.

Supplementary Figure 1 and 2

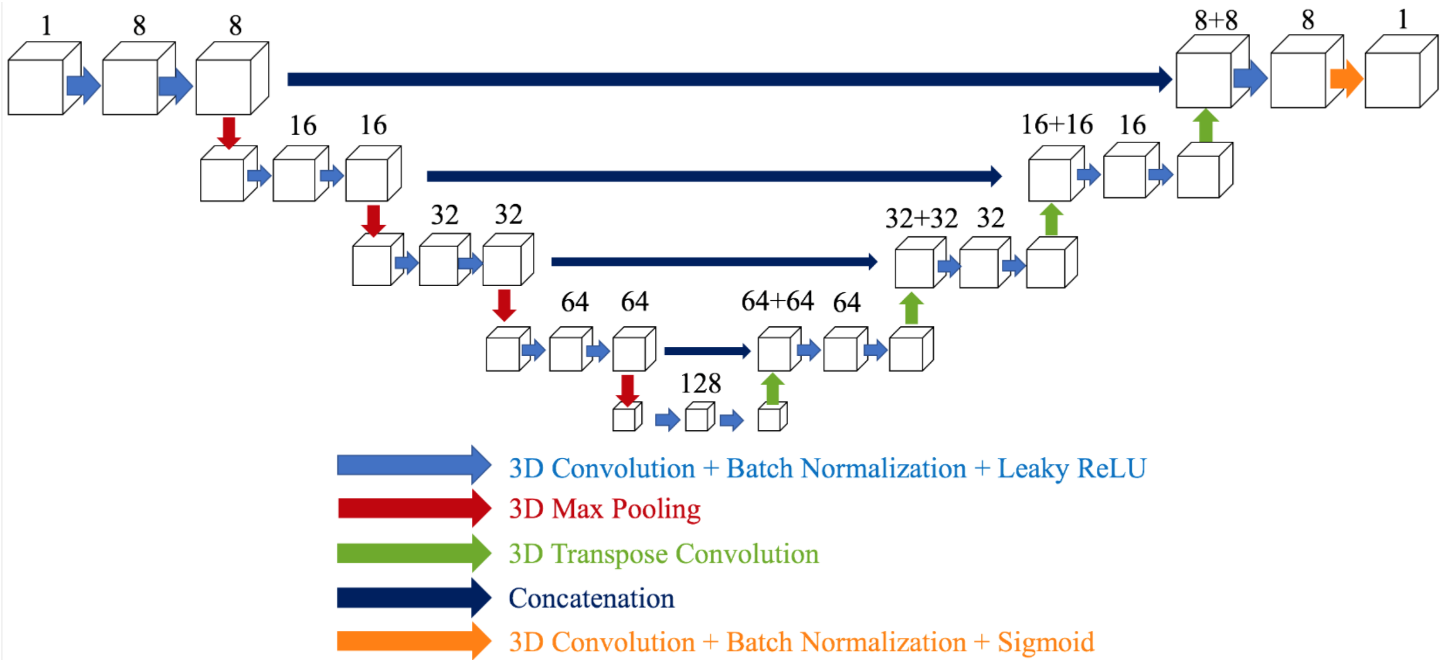

Supplementary Figure 1 – Architecture of the segmentation network of the DeepSynth CNN

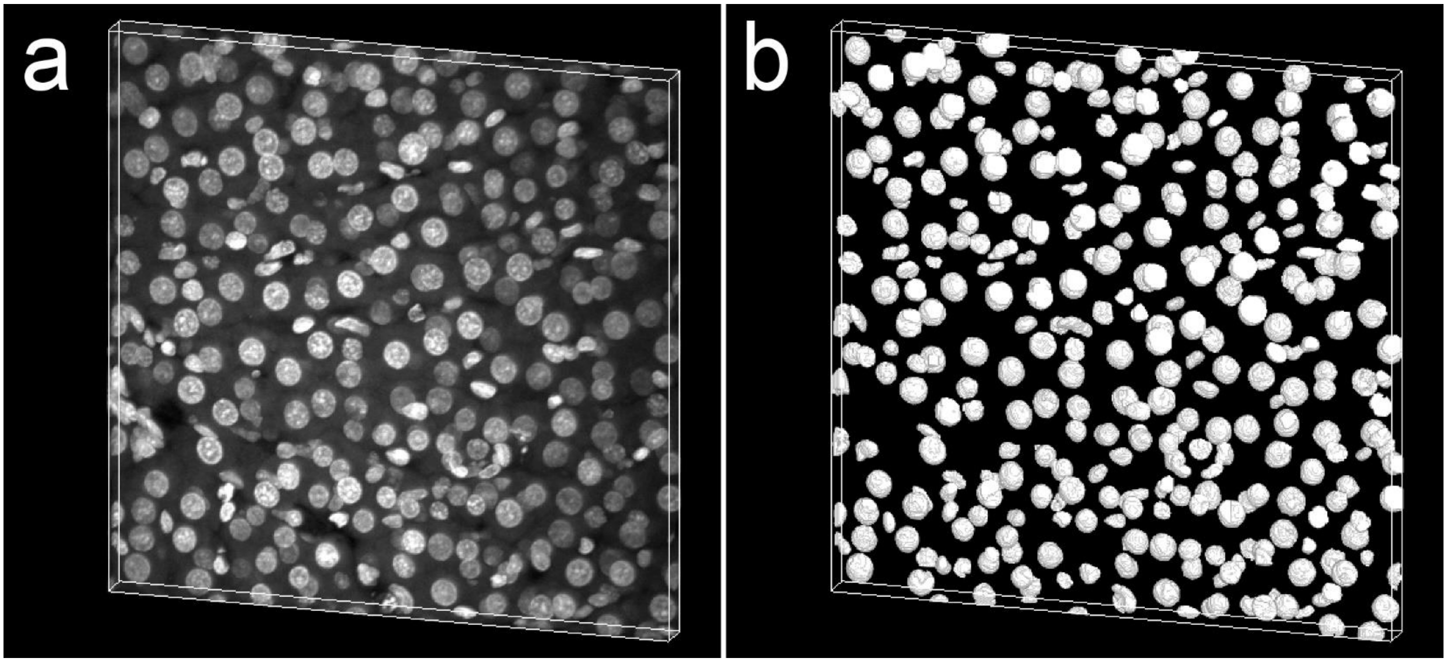

Supplementary Figure 2 – Three-dimensional ground-truth nuclei data. A - Maximum projection volume rendering of a 3D volume of images of nuclei in a fixed section of a rat kidney. B – Alpha-blending projection of a volume rendering of the corresponding hand-outlined, ground-truth image volume. Image volume collected from a field that was 256 microns across.

Legends to supplementary videos

Video 1 – Volume rendering of the three-dimensional image of cleared kidney shown in Figure 1

Video 2 – Figure 2: Comparison of DeepSynth with CellProfiler - Volume renderings of segmentation results obtained for a 130-162 micron deep section of the three-dimensional image of cleared kidney shown in Figure 3. Left to right – DeepSynth, original volume, CellProfiler, Cellprofiler after optimization

Video 3 – Figure 2: Comparison of DeepSynth with FARSIGHT - Volume renderings of segmentation results obtained for a 130-162 micron deep section of the three-dimensional image of cleared kidney shown in Figure 3. Left to right – DeepSynth, original volume, FARSIGHT, FARSIGHT after optimization

Video 4 – Figure 2: Comparison of DeepSynth with Squassh - Volume renderings of segmentation results obtained for a 130-162 micron deep section of the three-dimensional image of cleared kidney shown in Figure 3. Left to right – DeepSynth, original volume, Squassh, Squassh after optimization

Video 5 – Volume rendering of the three-dimensional image of cleared kidney shown in Figure 3

Video 6 – Figure 4: Comparison of DeepSynth with CellProfiler - Volume renderings of segmentation results obtained for a 31-50 micron deep section of the three-dimensional image of cleared kidney shown in Figure 3. Left to right – DeepSynth, original volume, CellProfiler, Cellprofiler after optimization

Video 7 – Figure 4: Comparison of DeepSynth with FARSIGHT - Volume renderings of segmentation results obtained for a 31-50 micron deep section of the three-dimensional image of cleared kidney shown in Figure 3. Left to right – DeepSynth, original volume, FARSIGHT, FARSIGHT after optimization

Video 8 – Figure 4: Comparison of DeepSynth with Squassh - Volume renderings of segmentation results obtained for a 31-50 micron deep section of the three-dimensional image of cleared kidney shown in Figure 3. Left to right – DeepSynth, original volume, Squassh, Squassh after optimization

Video 9 – Volume rendering of the three-dimensional image of rat liver tissue shown in Figure 5

Video 10 – Figure 6: Comparison of DeepSynth with CellProfiler - Volume renderings of segmentation results obtained for the three-dimensional image of rat liver tissue shown in Figure 5. Left to right – DeepSynth, original volume, CellProfiler, Cellprofiler after optimization

Video 11 – Figure 6: Comparison of DeepSynth with FARSIGHT - Volume renderings of segmentation results obtained for the three-dimensional image of rat liver tissue shown in Figure 5. Left to right – DeepSynth, original volume, FARSIGHT, FARSIGHT after optimization

Video 12 – Figure 6: Comparison of DeepSynth with Squassh - Volume renderings of segmentation results obtained for the three-dimensional image of rat liver tissue shown in Figure 5. Left to right – DeepSynth, original volume, Squassh, Squassh after optimization

Video 13 – Figure 7: Comparison of DeepSynth with CellProfiler - Volume renderings of segmentation results obtained for the three-dimensional image of mouse intestine. Left to right – DeepSynth, original volume, CellProfiler, Cellprofiler after optimization

Video 14 – Figure 7: Comparison of DeepSynth with FARSIGHT - Volume renderings of segmentation results obtained for the three-dimensional image of mouse intestine. Left to right – DeepSynth, original volume, FARSIGHT, FARSIGHT after optimization

Video 15 – Figure 7: Comparison of DeepSynth with Squassh - Volume renderings of segmentation results obtained for three-dimensional image of mouse intestine. Left to right – DeepSynth, original volume, Squassh, Squassh after optimization
